# The physiological effects of cardiac resynchronization therapy on aortic and pulmonary flow and dynamic and static components of systemic impedance

**DOI:** 10.1016/j.hroo.2021.05.007

**Published:** 2021-05-28

**Authors:** Baldeep S. Sidhu, Simon Claridge, Haotian Gu, Ye Li, Justin Gould, Bradley Porter, Mark K. Elliott, Vishal Mehta, Tom Jackson, Tiffany Patterson, Natalia Briceno, Jack Lee, Simon Redwood, Shaumik Adhya, Steven A. Niederer, Phil Chowienczyk, Christopher A. Rinaldi

**Affiliations:** ∗ School of Biomedical Engineering and Imaging Sciences, King’s College London, London, United Kingdom; † Guy’s and St Thomas’ Hospital, London, United Kingdom; ‡ British Heart Foundation Centre, King’s College London, London, United Kingdom; § Cardiovascular Department, King’s College London, London, United Kingdom; ‖ NIHR Biomedical Research Centre, School of Cardiovascular Medicine and Sciences, King’s College London, London, United Kingdom

**Keywords:** Aortic flow, Cardiac resynchronization therapy, Physiology, Pressure-volume loops, Pulmonary flow, Wave intensity analysis

## Abstract

**Background:**

Patients who improve following cardiac resynchronization therapy (CRT) have left ventricular (LV) remodeling and improved cardiac output (CO). Effects on the systemic circulation are unknown.

**Objective:**

To explore the effects of CRT on aortic and pulmonary blood flow and systemic afterload.

**Methods:**

At CRT implant patients underwent a noninvasive assessment of central hemodynamics, including wave intensity analysis (n = 28). This was repeated at 6 months after CRT. A subsample (n = 11) underwent an invasive electrophysiological and hemodynamic assessment immediately following CRT. CRT response was defined as reduction in LV end-systolic volume ≥15% at 6 months.

**Results:**

In CRT responders (75% of those in the noninvasive arm), there was a significant increase in CO (from 3 ± 2 L/min to 4 ± 2 L/min, *P =* .002) and LV dP/dt_max_ (from 846 ± 162 mm Hg/s to 958 ± 194 mm Hg/s, *P* = .001), immediately after CRT in those in the invasive arm. They demonstrated a significant increase in aortic forward compression wave (FCW) both acutely and at follow-up. The relative change in LV dP/dt_max_ strongly correlated with changes in the aortic FCW (*R*_*s*_ 0.733, *P* = .025). CRT responders displayed a significant reduction in afterload, and a decrease in systemic vascular resistance and pulse wave velocity acutely; there was a significant decrease in acute pulmonary afterload measured by the pulmonary FCW and forward expansion wave.

**Conclusion:**

Improved cardiac function following CRT is attributable to a combination of changes in the cardiac and cardiovascular system. The relative importance of these 2 mechanisms may then be important for optimizing CRT.

## Graphical abstract

**Figure:**
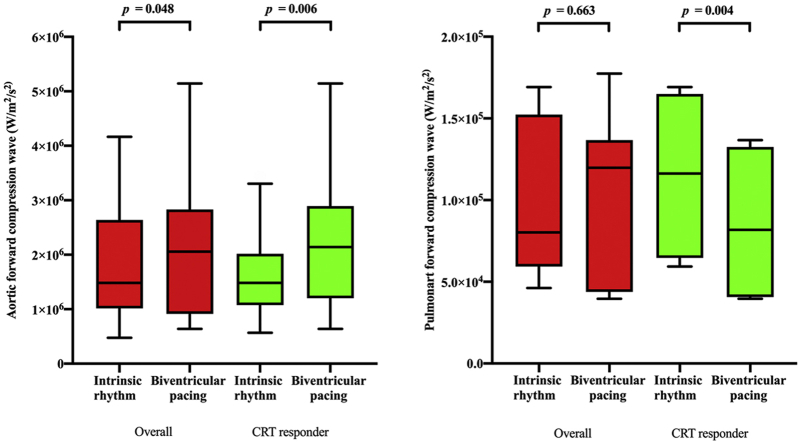

**Key Findings**

▪ In patients who improve following cardiac resynchronization therapy (CRT) there is evidence of ventricular resynchronization and reverse left ventricular (LV) remodeling. However, it is unknown whether there are concomitant changes in loading conditions on the left ventricle and/or changes in systemic impedance.
▪ In CRT responders, there is evidence of increased cardiac output and reduction in systemic vascular resistance.
▪ In CRT responders, there is an increase in aortic forward compression wave (FCW) and reduction in pulmonary FCW.
▪ There is a strong positive correlation between maximal rise in LV pressure and aortic FCW.
▪ In CRT responders there are significant changes to both the cardiac and vascular system. Principally, there is an increase in myocardial contractility and LV dP/dt_max_, and vascular changes with a resulting decrease in afterload. These combined changes lead to an increased cardiac output, stroke work, and mean arterial blood pressure.

## Introduction

Cardiac resynchronization therapy (CRT) improves symptoms and reduces mortality in symptomatic patients with left ventricular (LV) systolic impairment and electrical dyssynchrony, who are on optimal medical therapy.^1^ However, even in carefully selected cases approximately 30% of patients fail to benefit.^1^ In patients who improve following CRT there is evidence of ventricular resynchronization, reverse LV remodeling, increased cardiac output (CO), and increased external work achieved by the heart.^2^ It is unknown whether these physiological changes result purely from improved LV contraction, thus increasing CO, or whether there are concomitant changes in loading conditions on the left ventricle and/or changes in systemic impedance following CRT. Previous studies have shown that CRT is associated with improved right ventricular function and reduced systolic pulmonary pressures,^3^ but the mechanisms through which these effects are seen are not fully understood. In humans, it is understood that CRT improves coronary flow in the left anterior descending artery,^4^ but the effect of dynamic aortic and pulmonary pressure and flow changes on cardiac function requires further investigation. Wave intensity analysis enables the study of cardiovascular dynamics by representing pressure and velocity waveforms as successive wavefronts.^5^ The forward compression wave (FCW) measures the increase in pressure and flow through an artery and characterizes blood flow during early systole.^5^^,^^6^ The forward expansion wave (FEW) measures the decrease in pressure and flow in late systole. The backward compression wave (BCW) measures the increase in pressure and reduced flow through an artery and characterizes blood flow during mid-systole. These waves are present in both the systemic and pulmonary circulation.^6^^,^^7^ Determining whether there is a correlation between aortic flow and myocardial contractility helps understand how improved myocardial contractility seen with CRT may influence aortic flow in CRT responders and nonresponders.

Although CRT is known to increase CO, the effect on different components of afterload, namely systemic vascular resistance (SVR) and arterial stiffness, is not fully understood. Adjunctive medical therapy used in the treatment of heart failure alters afterload,^1^ and identifying whether CRT has additional effects on the vascular system is important in these patients. Aortic pulse wave velocity (PWV) is considered the gold standard for measuring central aortic stiffness and can be used to provide important prognostic information for a variety of conditions.^8^ A greater PWV is independently associated with incident clinical heart failure.^9^ Therefore, understanding whether CRT alters arterial hemodynamics has implications on potential therapeutic interventions.^10^

The purpose of this study was to explore the effects of CRT on aortic and pulmonary blood flow and afterload by comparing intrinsic rhythm with biventricular pacing. We hypothesized that response to CRT would be associated with cardiac changes, principally improved myocardial contractility and stroke work, and also changes in the cardiovascular system, which together would facilitate an increased mean arterial blood pressure. We measured wave intensity invasively and noninvasively and used pressure-volume loops to accurately assess LV hemodynamics.

## Methods

### Study design

The study was approved by the London Research Ethics Committee (11/LO/1232), all patients provided written informed consent for participation in this study, and the research in this study was conducted to the Helsinki Declaration guidelines on human research. The inclusion criteria involved patients with a guideline-directed indication for CRT.^11^ Patients were excluded if they were under 18 years old, pregnant, or unwilling to undergo noninvasive assessment at 6 months; and patients were also excluded from the invasive arm of the study if they had significant aortic valve disease or a contraindication to heparin. A quadripolar LV lead was placed at the basal lateral wall via the posterolateral or lateral coronary vein, wherever possible, and targeted to areas of latest electrical activation as assessed by the Q-LV time. The pacing vector that produced the narrowest QRS duration was chosen. Immediately following CRT, patients underwent an invasive and/or noninvasive assessment of cardiac function and pulmonary and systemic hemodynamics.

### Noninvasive assessment of aortic flow and arterial stiffness

This was performed on the same day following CRT and repeated at 6 months, using a pacing protocol described below and using a similar protocol to previously published work.^7^ Brachial blood pressure was measured by a validated oscillometric method (Omron 705CP; Omron Healthcare, Tokyo, Japan). Radial and carotid pressure waveforms were obtained using the SphygmoCor system (AtCor, West Ryde, Australia). Radial pressure was calibrated from the measured values of brachial systolic and diastolic pressure because these are assumed to be equal at both sites. Systolic pressure differs at the carotid and brachial sites, and therefore the carotid pressure was calibrated from the mean and diastolic pressure, which are similar at all 3 sites.^12^ Mean pressure is derived from radial pressure integrated over time. Femoral pressure waveforms were recorded by applanation tonometry using the SphygmoCor system. PWV was calculated from the transit time between the carotid to femoral pressure waveforms. The SphygmoCor device and transducer was used to record both pressure waveforms. The difference in the time of pulse arrival between these 2 sites was referenced to the R wave of the electrocardiogram and taken as the transit time. The path length was estimated from the distance between the sternal notch and femoral artery whereby the artery was applanated. PWV was then calculated as the path length divided by the transit time. Aortic flow was recorded from an echocardiographic apical 5-chamber view using continuous Doppler.

### Invasive protocol

Simultaneous invasive hemodynamic and electrophysiological measurements were performed immediately following successful CRT. Successful CRT was defined as evidence of biventricular pacing following LV lead placement and narrowing of the QRS duration. A pressure-volume loop conductance catheter (CD Leycom, Hengelo, Netherlands) was placed within the left ventricle and the tip of a 0.014-inch dual-pressure Doppler sensor wire (ComboWire 9500; Volcano Corp, San Diego, CA) within the ascending aorta for aortic flow and a pacing protocol undertaken. The pressure-Doppler wire was then re-sited in the main pulmonary artery for pulmonary flow and the pacing protocol repeated.

### Processing waveform data and PWV

Noninvasive ensemble averaged carotid pressures were used as a surrogate for ascending aortic pressure and, together with aortic flow velocity, were processed using MATLAB (MathWorks, Natick, MA) for wave intensity, pulse wave, and wave decomposition analysis.^7^

Invasive waveform data were processed using a similar protocol to previously published work.^2^^,^^4^ Data was imported into CardiacWaves (King’s College London, London, UK). Wave intensity analysis was performed using previously described methods.^4^^,^^13^ A polynomial filter was used to refine the derivatives of aortic/pulmonary pressure and velocity signals, using a Savitzky-Golay convolution method.^13^ The chosen 3–5 beats were gated to the R wave on the electrocardiogram, with ensemble averaging of the aortic/pulmonary pressure, average peak velocity, and heart rate.

**Figure 1:**
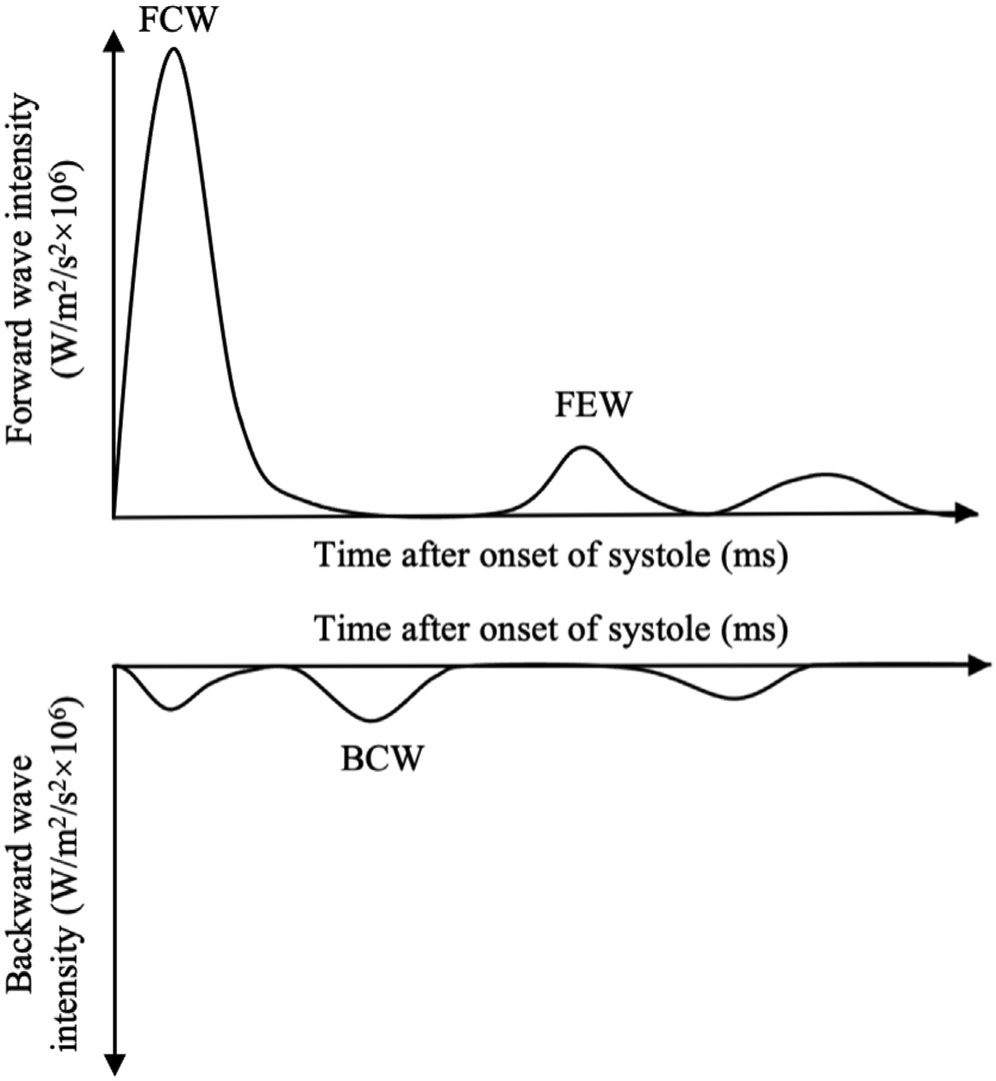
The predominant waves seen in aortic and pulmonary flow are displayed. The forward-traveling waves consist of the forward compression wave and forward expansion wave. The backward compression wave is the predominant backward-traveling wave. BCW = backward compression wave; FCW = forward compression wave; FEW = forward expansion wave.

Wave intensity (d*I*) was calculated from the time derivatives (dt) of ensemble-averaged aortic/pulmonary pressure (d*P*) and flow velocity (d*U*) as shown: d*I* = d*P*/dt × d*U*/dt.^5^^,^^13^ Corresponding forward and backward propagating waves were separated assuming a blood density of 1050 kg/m^3^ and estimating aortic/pulmonary wave speed using the sum of squares method.^13^ The peak energy carried by the 3 most prominent wave energies were analyzed and recorded in this manuscript; the FCW, FEW, and BCW (Figure 1).

### Processing invasive hemodynamic data

Simultaneous LV pressure and volume were measured and volume calibration was performed off-line post data acquisition using 3-dimensional echocardiography to obtain LV ejection fraction, LV end-diastolic volume, and LV end-systolic volume. Hemodynamic data were recorded on Conduct NT (CD Leycom). Data were sampled at 250 Hz and exported into SimpleWires (King’s College London). At least 10 consecutive cycles were selected, with ensemble average of at least 5 beats for analysis. The resulting pressure-volume loop was exported to provide invasive hemodynamic data.^14^

### Pacing protocol

Biventricular pacing at baseline heart rates was compared with intrinsic rhythm at baseline heart rates. Measurements for patients in sinus rhythm were made in AAI mode, atrial fibrillation in VVI mode, and complete heart block in DDD mode. Baseline heart rates were 10 beats/min above the patient’s intrinsic rate or at 70 beats/min in patients with complete heart block.^4^ The atrioventricular delay was set to 120 ms and simultaneous ventricular activation.

### Definition of CRT responders

Patients were defined as CRT responders if they had a reduction in LV end-systolic volume ≥15% at 6-month follow-up.^1^^,^^15^

### Statistical analysis

Discrete data are presented as n values with corresponding percentages in parentheses and continuous data as means ± standard deviation. Responses in the same participants at different pacing settings were compared using a paired 2-sided Student *t* test for normally distributed data and Wilcoxon signed rank test for non-normally distributed data. A 2-sided *P* value < .05 was considered statistically significant. Statistical analyses were performed using Prism (Version 8; GraphPad Software Inc, La Jolla, CA) and SPSS (Version 25; IBM, Armonk, NY).

## Results

**Figure 2:**
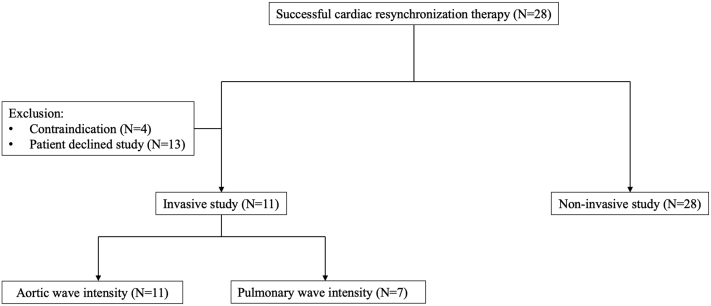
Patient recruitment into noninvasive and invasive studies.

**Figure 3:**
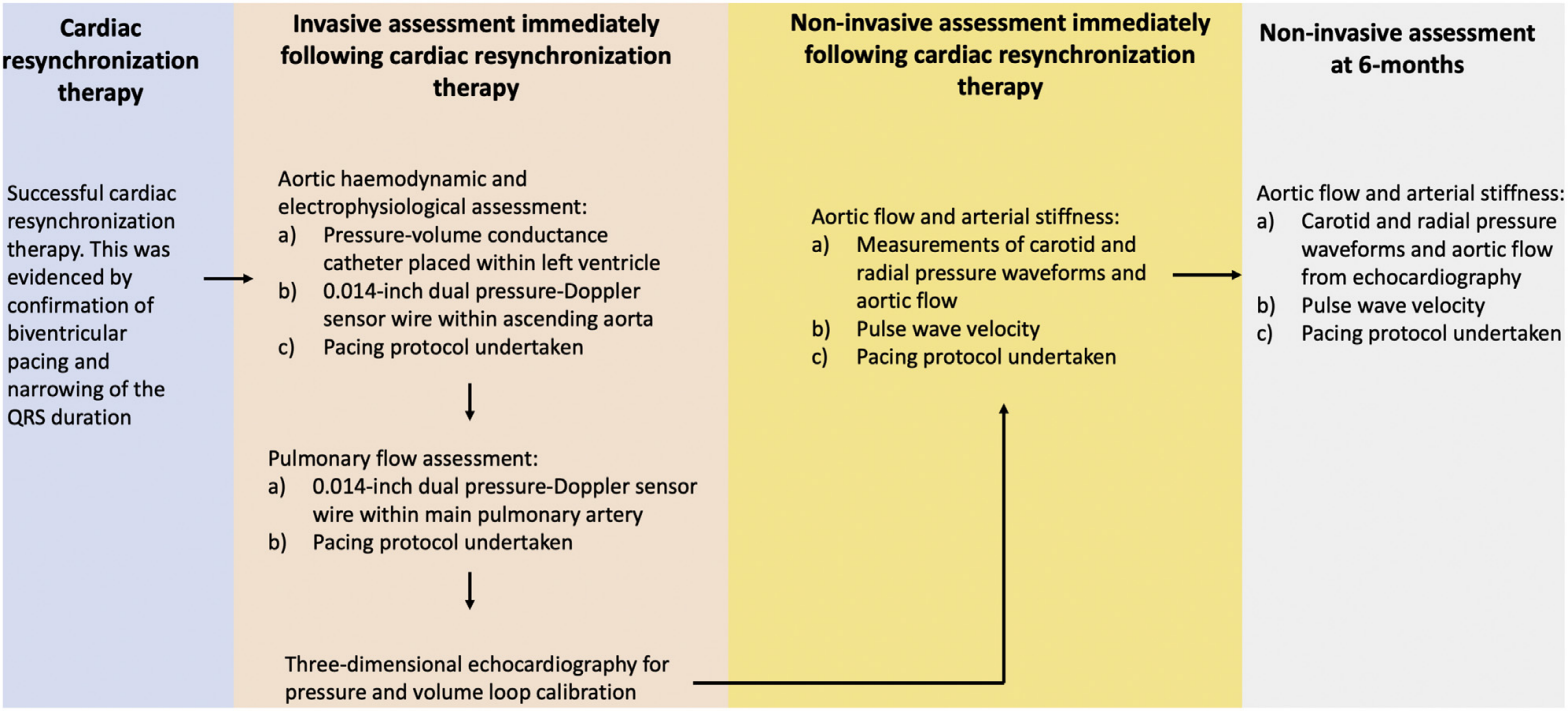
Flowchart of invasive and noninvasive arms of the study.

Patient recruitment is shown in Figure 2, a flowchart of the study in Figure 3, and baseline demographics in Table 1. All 28 patients underwent successful CRT, with a quadripolar LV lead placed in a lateral or posterolateral vein in 25 (89.3%) patients. All patients survived to 6-month follow-up and had >99% biventricular pacing delivery confirmed at 6 months. Supplemental Appendix A provides a subgroup analysis of patients who were only in sinus rhythm and left bundle branch block.

**Table 1:** Baseline patient demographics

Variable	Noninvasive protocol				Invasive protocol			
	Overall (N = 28)	CRT responders (N = 21)	CRT nonresponders (N = 7)	*P* value†	Overall (N = 11)	CRT responders (N = 7)	CRT nonresponders (N = 4)	*P* value†
Age, ± SD (years)	72.9 ± 8	73.6 ± 7	70.8 ± 10.8	.444	68.1 ± 9.1	68.6 ± 7.9	67.3 ± 12.3	.705
Male, n (%)	20 (71.4)	14 (66.7)	6 (85.7)	.633	9 (81.8)	6 (85.7)	3 (75)	1.000
Ischemic etiology, n (%)	15 (53.6)	10 (47.6)	5 (71.4)	.396	5 (45.5)	2 (28.6)	3 (75)	.242
NYHA functional class, ± SD	2.8 ± 0.7	2.8 ± 0.8	2.9 ± 0.4	.917	2.5 ± 0.8	2.4 ± 1	2.8 ± 0.5	.477
Sinus rhythm, n (%)	19 (67.9)	14 (66.7)	5 (71.43)	1.000	8 (72.7)	5 (71.4)	3 (75.0)	1.000
LBBB, n (%)	24 (85.7)	20 (95.2)	4 (57.1)	.038	7 (63.6)	5 (71.4)	2 (50)	.576
QRS duration, ± SD	158 ± 19	158 ± 20	157 ± 17	.860	151 ± 18	150 ± 20	152 ± 16	.861
LVEF, ± SD	30 ± 8	30 ± 7	29 ± 10	.761	27 ± 9	26 ± 5	28 ± 14	.755
LVEDV, ± SD	164 ± 58	157 ± 61	185 ± 41	.140	196 ± 68	197 ± 85	195 ± 32	.963
LVESV, ± SD	117 ± 50	113 ± 53	130 ± 39	.172	143 ± 60	146 ± 71	137 ± 44	.818

CRT = cardiac resynchronization therapy; LBBB =left bundle branch block; LVEDV =left ventricular end-diastolic volume; LVEF = left ventricular ejection fraction; LVESV = left ventricular end-systolic volume; NYHA = New York Heart Association.

† Comparison between CRT responders and CRT nonresponders.

### Noninvasive protocol

Overall, 21 (75.0%) patients were CRT responders and 7 (25.0%) CRT nonresponders. Patient demographics include a mean age of 72.9 ± 8.0 years; 53.6% of patients had ischemic heart disease; 85.7% had left bundle branch block, with a mean QRS duration of 158 ± 19ms and a severely reduced LV ejection fraction of 30% ± 8%.

In CRT responders at 6 months, biventricular pacing resulted in a significant increase in the systolic blood pressure (106.8 ± 18.4 vs 97.9 ± 18.3 mm Hg; *P* = .015), mean arterial pressure (84.2 ± 12.3 vs 77.7 ± 13.7 mm Hg; *P* = .046), and central pulse pressure (38.8 ± 15.2 vs 35.1 ± 14.9 mm Hg; *P* = .031) but not the diastolic blood pressure (68.0 ± 10.7 vs 62.8 ± 13.0 mm Hg; *P* = .083). There was no significant difference in CRT nonresponders at 6 months in the systolic blood pressure, mean arterial pressure, or central pulse pressure.

### Noninvasive aortic wave intensity at baseline heart rates

**Figure 4:**
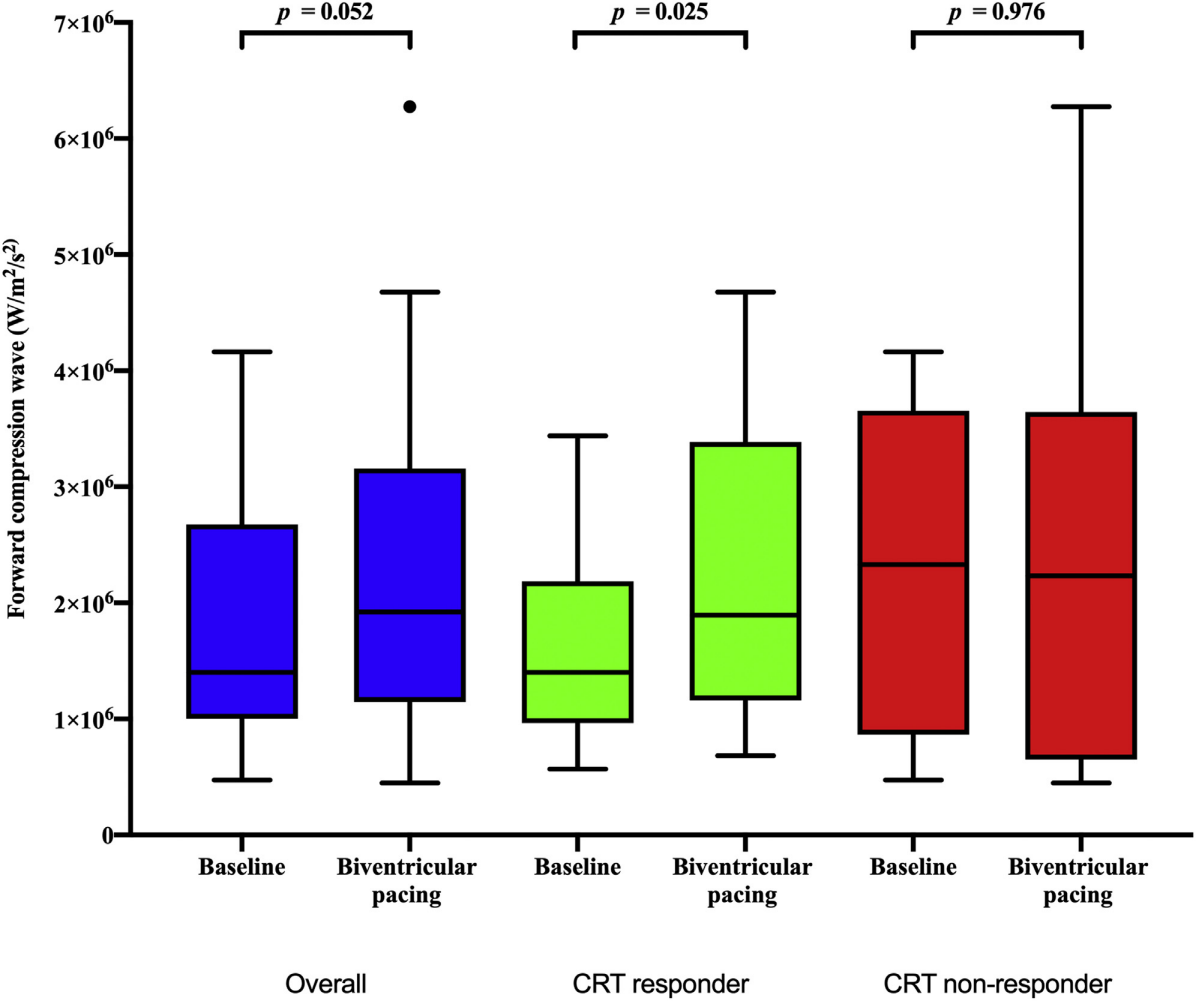
Box-and-whisker plot showing the noninvasive aortic wave intensity at baseline rates before cardiac resynchronization therapy and at 6 months with biventricular pacing in different patient groups. Tukey whiskers have been used to represent the data by displaying the box, consisting of the median and upper and lower quartiles, and the whiskers, consisting of the maximum and minimum value, followed by any outlying patient data, represented by a dot.

In CRT responders, biventricular pacing compared with intrinsic rhythm resulted in an immediate increase in the FCW (2.1 [1.3–2.8] vs 1.4 [1.1–2.0] W/m^2^/s^2^ × 10^6^; *P* = .006) but not the FEW and BCW (Figure 4 and Table 2). There was no significant difference in the timing of the FCW wave (35.7 vs 37.9 ms; *P* = .255). These findings were maintained at 6 months, with a significant increase in the FCW (*P* = .025). These effects were not seen in CRT nonresponders.

**Table 2:** Noninvasive aortic wave intensity analysis after CRT

Variable	Overall (N = 28)	CRT responders (N = 21)	CRT nonresponders (N = 7)
Forward compression wave (W/m^2^/s^2^×10^6^)			
Baseline before CRT	1.4 [1.1–2.5]	1.4 [1.1–2.0]	2.3 [1.1–3.5]
Acutely following CRT	2.1 [1–2.8]	2.1 [1.3–2.8]	0.8 [0.8–2]
6-month follow-up	1.9 [1.1–2.9]	1.9 [1.2–3.1]	1.9 [0.9–2.6]
*P* value of intrinsic vs acute biventricular pacing following CRT	.048	.006	.600
*P* value of intrinsic vs biventricular pacing at 6-months	.052	.025	.976
*P* value of intrinsic vs biventricular pacing at 6-months	.977	.711	.345
Forward expansion wave (W/m^2^/s^2^×10^6^)			
Baseline before CRT	0.4 [0.3–0.7]	0.5 [0.3–0.6]	0.2 [0.1–0.9]
Acutely following CRT	0.4 [0.2–0.5]	0.4 [0.3–0.7]	0.2 [0.2–0.3]
6-month follow-up	0.4 [0.2–0.6]	0.4 [0.3–0.6]	0.3 [0.2–0.7]
*P* value of intrinsic vs acute biventricular pacing following CRT	.476	.794	.345
*P* value of intrinsic vs biventricular pacing at 6-months	.784	.711	.686
*P* value of biventricular pacing acutely vs 6-months	.429	.845	.250
Backward compression wave (W/m^2^/s^2^×10^6^)			
Baseline before CRT	0.2 [0.1–0.5]	0.2 [0.1–0.6]	0.1 [0.1–0.4]
Acutely following CRT	0.3 [0.1–0.5]	0.4 [0.2–0.6]	0.1 [0.1–0.1]
6-month follow-up	0.2 [0.1–0.3]	0.2 [0.1–0.3]	0.1 [0.1–0.2]
*P* value of intrinsic vs acute biventricular pacing following CRT	.753	.872	.345
*P* value of intrinsic vs biventricular pacing at 6-months	.260	.433	.394
*P* value of biventricular pacing acutely vs 6-months	.334	.300	.532

Results are presented as median [interquartile range] for ease of comparison.

CRT = cardiac resynchronization therapy.

### PWV

In CRT responders, biventricular pacing resulted in a significant reduction in the PWV acutely (0.5 ± 1.2 m/s; *P* = .021) and a nonsignificant reduction at follow-up (0.7 ± 1.6 m/s; *P =* .086) when compared with intrinsic rhythm. There were no significant differences in changes from baseline in PWV in CRT nonresponders.

### Invasive protocol

The invasive study was used to validate the major findings of the noninvasive study. Eleven patients underwent an invasive protocol with 11 aortic and 7 pulmonary electrophysiology recordings. Acquisition of pressure-volume loop data was attempted in all cases but was successful in 9 patients, since there was significant interference during data collection in 2 patients, preventing reliable recordings. There were no acute complications arising from this study.

Overall, 7 (63.6%) patients met the study definition for CRT response. Patient demographics include a mean age of 68.1 ± 9.1 years, QRS duration of 151 ± 18 ms, and severely impaired LV ejection fraction of 27% ± 9% (Table 1). In CRT responders, biventricular pacing resulted in a significant increase in the CO (4 ± 2 vs 3 ± 2 L/min; *P* = .002) and reduction in SVR (26 ± 10 vs 44 ± 26 mm Hg/min/L; *P* = .040) (Table 3). There was no significant difference in CO or SVR in CRT nonresponders. All CRT responders showed an acute hemodynamic improvement in LV dP/dt_max_ >10%, which was not seen in any of the nonresponders.

**Table 3:** Invasive hemodynamic data

Variable	Overall (N = 9)			CRT responder (N = 6)			CRT nonresponder (N = 3)		
	Intrinsic	Biventricular pacing	*P* value^†^	Intrinsic	Biventricular pacing	*P* value^†^	Intrinsic	Biventricular pacing	*P* value^†^
MAP, mm Hg	87 ± 20	88 ± 19	.731	89 ± 24	89 ± 23	.741	83 ± 12	84 ± 6	.875
SVR, mm Hg/min/L	35 ± 25	23 ± 10	.051	44 ± 26	26 ± 10	.040	17 ± 2	19 ± 9	.760
LVEDV, mL	230 ± 71	234 ± 76	.577	229 ± 85	236 ± 84	.365	232 ± 48	232 ± 70	.984
LVESV, mL	184 ± 67	173 ± 64	.069	192 ± 80	182 ± 76	.227	168 ± 39	155 ± 28	.285
SV, mL	46 ± 26	61 ± 35	.015	37 ± 23	53 ± 27	.002	64 ± 25	77 ± 51	.497
CO, L/min	3 ± 2	4 ± 2	.028	3 ± 2	4 ± 2	.002	4 ± 1	4 ± 1	.260
SW, mm Hg/mL	4988 ± 2627	6327 ± 3035	.007	4532 ± 2734	6198 ± 2770	.004	5899 ± 2653	6586 ± 4186	.520
EDPVR, mm Hg/mL	0.1 ± 0.1	0.1 ± 0.0	.750	0.1 ± 0.1	0.1 ± 0.0	.249	0.1 ± 0.0	0.1 ± 0.0	.593
ESPVR, mm Hg/mL	0.7 ± 0.2	0.8 ± 0.2	.188	0.8 ± 0.3	0.8 ± 0.2	.888	0.7 ± 0.1	0.7 ± 0.2	.634
LV dP/dt_max,_, mm Hg/s	846 ± 130	912 ± 169	.045	846 ± 162	958 ± 194	.001	846 ± 40	820 ± 40	.615
LV dP/dt_min_, mm Hg/s	830 ± 105	856 ± 133	.507	864 ± 95	921 ± 94	.006	760 ± 102	726 ± 105	.794

Results are presented as mean ± standard deviation.

CO = cardiac output; EDPVR = end-diastolic pressure-volume relationship; ESPVR = end-systolic pressure-volume relationship; LV = left ventricular; LVEDV = left ventricular end-diastolic volume; LVESV = left ventricular end-systolic volume; MAP = mean arterial pressure; SV = stroke volume; SVR = systemic vascular resistance; SW = stroke work.

### Invasive aortic wave intensity and correlation between aortic flow and myocardial contractility

In CRT responders, biventricular pacing compared with intrinsic rhythm resulted in a significant increase in the FCW acutely (8.3 [4.4–8.4] vs 4.8 [3.0–7.0] W/m^2^/s^2^ × 10^5^; *P* = .023) (Supplemental Appendix B) and shorter time to peak FCW (50.0 vs 55.0 ms; *P* = .020). These effects were not seen in CRT nonresponders. The relative change in LV dP/dt_max_ strongly correlated with the change in aortic FCW (R_s_ 0.733; *P* = .025).

### Invasive pulmonary wave intensity following CRT

There were 4 (57.1%) CRT responders and 3 (42.9%) CRT nonresponders who underwent pulmonary flow assessment (Supplemental Appendix B). In CRT responders, biventricular pacing resulted in a significant reduction in the FCW (0.8 [0.4–1.2] vs 1.2 [0.8–1.6] W/m^2^/s^2^ × 10^5^; *P* = .004) and FEW (*P* = .030) (Supplemental Appendix B). Biventricular pacing resulted in a significantly longer time to the peak FCW (72.5 vs 47.5 ms; *P* = .009) and FEW (268.8 vs 212.5 ms; *P* = .014). These changes were not seen in CRT nonresponders.

## Discussion

To our knowledge, this is the first study to comprehensively examine the effects of biventricular pacing on aortic and pulmonary flow and determine its effects on SVR and PWV. Although wave intensity analysis has traditionally been measured invasively, we sought to determine the effects of CRT both acutely and chronically, requiring us to use a combination of invasive and noninvasive measurements. We found the following effects in CRT responders:

(1) Significant increase in CO and decrease in SVR; there was a significant reduction in the PWV acutely and a nonsignificant reduction chronically
(2) Significant increase in the aortic FCW both acutely and chronically
(3) Strong positive correlation between maximal rise in LV pressure and aortic FCW
(4) Significant reduction in the pulmonary FCW and FEW

This study demonstrates that in CRT responders there are significant changes to both the cardiac and vascular system. Principally, there is an increase in myocardial contractility and LV dP/dt_max_, and vascular changes with a resulting decrease in afterload. These combined changes lead to an increased CO, stroke work, and mean arterial blood pressure.

### Effect of CRT on aortic pressure/flow waves

The heart is part of an integrated system. Changes in cardiac contractility and perfusion are affected by preload and afterload, both of which dynamically respond to changes in cardiac function. The effects of CRT on coronary flow have previously been investigated,^2^^,^^4^ and they demonstrate that biventricular pacing increases coronary flow in the left anterior descending artery by increasing the backward expansion wave and homogenizing wave timings that determine flow in the left anterior descending and circumflex arteries. The effects of CRT on aortic flow are not well described. Fok and colleagues^7^ showed in hypertensive patients that dobutamine increased the FCW by a greater proportion than in normotensive patients. Dobutamine partly exerts its effects through improving myocardial contractility, and this improved contractility is evident in CRT responders. The current study demonstrates that in CRT responders there is a significant increase in the FCW both acutely and chronically. The relative change in acute LV pressure strongly correlated with the FCW at baseline heart rates, suggesting that as LV pressure increases, from improved myocardial contractility, so does aortic forward flow in early systole. The aortic forward flow can only increase if systemic impedance does not rise in parallel with the rise in LV pressure. When measured invasively, the timing to the peak FCW occurred significantly earlier in the cardiac cycle, which may enable longer diastolic filling and improved cardiac function.

### Effect of CRT on pulmonary pressure/flow waves

The effect of CRT on pulmonary wave intensity, to our knowledge, has not been previously described. In an invasive study of 31 patients investigating pulmonary flow, patients with pulmonary arterial hypertension and chronic thromboembolic pulmonary hypertension were shown to have a significantly higher FCW and FEW compared with control subjects.^6^ Severe LV systolic impairment is a common cause of postcapillary pulmonary hypertension. We demonstrated that in patients with severe LV systolic impairment who respond with CRT, biventricular pacing resulted in a significant reduction in the FCW and FEW. The time to the peak FCW and FEW was also significantly longer, in keeping with biventricular pacing, allowing for more effective LV relaxation and filling, thereby increasing preload, which in turn increases CO, evidenced by a significant increase in the LV dP/dt_min_.

### Effect of CRT on afterload

The effect of CRT on afterload has not been comprehensively investigated, to our knowledge. The 2 major components of afterload or impedance are PWV and SVR.^16^ PWV determines the characteristic impedance, which is afterload when pressure and flow are rapidly changing at the beginning of systole. However, SVR is the steady-state afterload. Importantly, a greater aortic FCW can only generate greater flow if impedance remains constant or falls. In the current manuscript, we have shown that CRT responders have a significant increase in FCW with a concomitant reduction in SVR and PWV acutely and nonsignificant reduction in PWV at follow-up. Several studies have shown that increased arterial blood pressure is associated with an increase in PWV.^16^ Our findings are in stark contrast to these studies, where we have shown that CRT responders demonstrate a rise in mean arterial blood pressure; however, this was not associated with a rise in PWV. Faconti and colleagues^10^ found a significant reduction in mean arterial pressure despite an increase in PWV after using lower-limb venous occlusion devices. They postulated that these findings were explained by sympathetic activation leading to increased vascular smooth muscle. Our findings could be explained by the increased FCW seen with biventricular pacing leading to greater pulsatility and CO, which resulted in decreased baroreceptor activation of the sympathetic nervous system owing to a higher pulse pressure and decreased activation of the renin-angiotensin-aldosterone system.

### Clinical perspective

This study offers new explanations as to how CRT may exert its benefits in heart failure patients. Understanding the role of the cardiovascular system on CRT response supports considering the cardiovascular system as a whole in CRT patient selection. Therefore, it is important to examine both the cardiac and systemic hemodynamics to understand who will respond best to CRT and how to optimize response. It is possible, for example, that those who show benefit in cardiac mechanics but not in systemic vascular responses could benefit from additional vasodilator therapy.

### Limitations

The study size was small because patients were asked to undergo a rigorous and lengthy invasive and noninvasive protocol; therefore, the results may not be generalizable to the whole CRT population. However, our sample size is in keeping with other published studies relating to invasive wave intensity analysis.^2^^,^^4^ The invasive protocol carried additional procedural risks and therefore is unlikely to be used in routine clinical practice, although the noninvasive protocol could be adopted. Both groups were matched in terms of sex, etiology, presence of left bundle branch block, QRS duration, and LV ejection fraction. The confidence intervals for hemodynamic and electrophysiology data in CRT nonresponders was wide owing to a small cohort, and consequently we are unable to speculate why they have failed to improve. During the pacing protocol we fixed the patient’s heart rate to control for the effect of changes in chronotropy can cause to inotropy, but it should be noted that this will prevent reflex heart rate regulation to alterations in inotropy. No variation in atrioventricular delay was assessed in the current study owing to the complexity of the protocol, and therefore we could not study the changes in acute hemodynamics that may have occurred with atrioventricular or ventricular optimization. Defining CRT response is heterogenous and can be based on hard and/or soft endpoints.^1^ The aim of this study was to further investigate the physiological effects of CRT on the cardiac and cardiovascular system, and therefore we used patients who displayed evidence of LV remodeling to define CRT response. Although the present study suggests an increased FCW in CRT responders, we are unable to provide a cut-off value for predicting response. Further studies with a larger sample size will be needed to determine whether this is possible. Furthermore, the study was underpowered to determine whether changes in FCW were different in patients with non–left bundle branch block and in CRT nonresponders whether the FCW worsens or remains static with biventricular pacing*.* The sample size was too small to draw reliable conclusions from the effect of rhythm (ie, sinus rhythm vs atrial fibrillation) alone and optimal pre-load is not possible for patients in atrial fibrillation.

## Conclusion

This study demonstrates that response to CRT is characterized by an increased FCW, due to increased cardiac contractility, with a reduction in both dynamic (PWV) and steady-state components (SVR) of afterload that results in an increased CO. Therefore, both cardiac and systemic vascular responses determine response to CRT, which may be particularly important in optimizing therapy and informing patient selection.

## Funding Sources

Wellcome/EPSRC Centre for Medical Engineering [WT203148/Z/16/Z] supported this study.

## Disclosures

B.S. Sidhu is funded by NIHR and has received speaker fees from EBR systems, outside the submitted work. H. Gu is supported by the NIHR ICA Clinical Lectureship, UK (ICA-CL-2018-04-ST2-012), outside the submitted work. H. Gu and P. Chowienczyk are supported by BHF Project Grant (PG/19/23/34259), outside the submitted work. J. Gould is funded from Rosetrees Trust/Abbott. B. Porter, M. Elliott, and V. Mehta are funded by Abbott. S. Adhya received funding from Abbott/Medtronic/Bayer. C.A. Rinaldi receives research fees from Abbott/Medtronic/Boston Scientific/MicroPort.

## Authorship

All authors attest they meet the current ICMJE criteria for authorship.

## Patient Consent

All patients provided written informed consent for participation in this study.

## Ethics Statement

The research in this study was conducted according to the Helsinki Declaration guidelines on human research. The study was approved by the London Research Ethics Committee (11/LO/1232).
