# Supplementary material for: The physiological effects of cardiac resynchronization therapy on aortic and pulmonary flow and dynamic and static components of systemic impedance
Source: Heart Rhythm O2. 2021 May 28;2(4):365–73. doi: 10.1016/j.hroo.2021.05.007 (PMC8369303; doi:10.1016/j.hroo.2021.05.007)
Supplement: Supplemental Appendix A [file mmc1.docx]

Appendix A

The results of patients in sinus rhythm and left bundle branch block are included in this section. Baseline demographics for the invasive and non-invasive study are provided in *Table B.1.* All 15 patients underwent successful CRT with a quadripolar LV lead placed in a lateral or posterolateral vein in 13 (86.7%) patients. All patients survived to 6-month follow-up and had >99% biventricular pacing delivery confirmed at 6 months.

***Non-invasive protocol***

Overall, 11 (73.3%) patients were CRT responders and 4 (26.7%) CRT non-responders. Patient demographics include a mean age of 69.9 ± 7.2 years, 46.7% had ischemic heart disease, with a mean QRS duration of 156 ± 18ms and a severely reduced LV ejection fraction of 31±6%.

*Non-invasive aortic wave intensity at baseline heart rates*

In CRT responders, biventricular pacing compared with intrinsic rhythm resulted in a significant increase in the FCW (2.7[1.8-3.8] vs. 1.9[1.3-2.5] W/m^2^/s^2^×10^6^*;P*=0.047) but not the FEW (0.5[0.3-0.8] vs. 0.3[0.3-0.6] W/m^2^/s^2^×10^6^*;P*=0.120) nor BCW (0.4[0.1-0.7] vs. 0.2[0.1-0.3] W/m^2^/s^2^×10^6^*;P*=0.123) *(Table B.2)*. There was no significant difference in the timing of the waves; FCW (35.2±4.1 vs. 36.7±5.3ms;*P*=0.414), FEW (205.1±22.8 vs. 222.7±25.4ms;*P*=0.058) and BCW (118.2±85.0 vs. 93.8±34.2ms;*P*=1.000). These findings were maintained at 6-months, with a significant increase in the FCW (2.8[2.7-3.2]-1.9[1.3-2.5] W/m^2^/s^2^×10^6^;*P*=0.026).

*PWV*

In CRT responders, biventricular pacing resulted in a non-significant reduction in the PWV acutely (0.2±1.1m/s;*P*=0.548) and significant reduction at follow-up (1.0±1.0m/s;*P=*0.021) when compared with intrinsic rhythm. There were no significant differences in acute or chronic PWV in CRT non-responders.

***Invasive protocol***

Overall, 8 patients underwent aortic and 6 pulmonary electrophysiology recordings. 5 (62.5%) patients met the study definition for CRT response and 3 (37.5%) patients were CRT non-responders. Patients demographics include a mean age of 66.1 ± 7.5 years, 37.5% had ischemic heart disease, with a mean QRS duration of 155 ± 18ms and a severely reduced LV ejection fraction of 30 ± 6%. Hemodynamic data following CRT is provided in *Table B.3.* In CRT responders, biventricular pacing compared with intrinsic rhythm resulted in a significant increase in the CO (5±3 vs. 3±2L/min;*P*=0.018) and reduction in SVR (23±8 vs. 37±17mmHg min/L;*P*=0.049). There was no significant difference in CO nor SVR in CRT non-responders. All CRT responders showed an acute hemodynamic improvement in LV dP/dt_max_ > 10% which was not seen in any of the non-responders.

*Invasive aortic wave intensity following CRT*

In CRT responders, biventricular pacing compared with intrinsic rhythm resulted in a significant increase in the FCW (7.8[3.4-8.4] vs. 4.0[2.0-6.7] W/m^2^/s^2^×10^5^;*P* = 0.031) and a significantly shorter time to the peak FCW (50.0 vs. 55.0ms;*P*=0.020).

*Correlation between aortic flow and myocardial contractility*

At baseline heart rates, the relative change in LV dP/dt_max_ strongly correlated with the change in aortic FCW (*R_s_* 0.786;*P*=0.036).

*Invasive pulmonary wave intensity following CRT*

There were 3 CRT responders and 3 CRT non-responders who underwent pulmonary flow assessment*.* In CRT responders, biventricular pacing resulted in a significant reduction in the FCW (0.4[0.4-0.9] vs. 0.8[0.7-1.2] W/m^2^/s^2^×10^5^;*P*=0.028) but not the FEW (0.1[0.1-0.2] vs. 0.4[0.3-0.5] W/m^2^/s^2^×10^5^;*P*=0.077) nor BCW (0.01[0.01-0.01] vs. 0.05[0.04-0.07] W/m^2^/s^2^×10^5^;*P*=0.577). Biventricular pacing resulted in a significantly longer time to the peak FCW (72.5 vs. 47.5ms;*P*=0.009) and FEW (268.8 vs. 212.5ms;*P*=0.014) but not the BCW (140.0 vs. 146.3ms;*P*=1.000). These changes were not seen in CRT non-responders.

**Table A.1.** Baseline patient demographics

| **Variable** | **Non-invasive protocol** | | | | **Invasive protocol** | | | |
| --- | --- | --- | --- | --- | --- | --- | --- | --- |
|  | **Overall**  **(N=15)** | **CRT responder**  **(N=11)** | **CRT non-responder**  **(N=4)** | ***P*-value^†^** | **Overall**  **(N=8)** | **CRT responder**  **(N=5)** | **CRT non-responder**  **(N=3)** | ***P*-value^†^** |
| Age, ±SD | 69.9±7.2 | 72.0±6.7 | 63.9±5.6 | 0.050 | 66.1±7.5 | 69.0±8.4 | 61.1±1.2 | 0.168 |
| Male, N(%) | 9(60) | 4(54.6) | 4(75) | 0.604 | 6(75) | 4(80) | 2(66.7) | 1.000 |
| Ischemic aetiology, N(%) | 7(46.7) | 5(45.5) | 2(50) | 1.000 | 3(37.5) | 1(20) | 2(66.7) | 0.464 |
| NYHA functional class, ±SD | 2.7±0.7 | 2.7±0.8 | 2.8±0.5 | 0.949 | 2.7±0.6 | 2.8±0.8 | 2.8±0.7 | 0.818 |
| QRS duration, ±SD | 156±18 | 158±18 | 150±17 | 0.423 | 155±18 | 156±20 | 153±19 | 0.825 |
| LVEF, ±SD | 31±6 | 31±6 | 33±5 | 0.463 | 30±6 | 27±3 | 35±5 | 0.300 |
| LVEDV, ±SD | 150±45 | 141±43 | 176±46 | 10.96 | 172±48 | 160±53 | 193±39 | 0.398 |
| LVESV, ±SD | 103±35 | 99±36 | 114±35 | 0.502 | 119±37 | 118±41 | 122±38 | 0.904 |

^†^Comparison between CRT responders and CRT non-responders

CRT,cardiac resynchronization therapy; LBBB,left bundle branch block; LVEDV,left ventricular end-diastolic volume; LVEF,left ventricular ejection fraction; LVESV,left ventricular end-systolic volume; NYHA,New York Heart Association

**Table A.2.** Non-invasive aortic wave intensity analysis after CRT

| **Variable** | **Overall (N=15)** | **CRT responders (N=11)** | **CRT non-responders (N=4)** |
| --- | --- | --- | --- |
| **Forward compression wave (W/m^2^/s^2^×10^6^)** |  |  |  |
| Baseline before CRT | 1.9[1.3-2.7] | 1.9[1.3-2.5] | 3.3[1.9-3.4] |
| Acutely following CRT | 2.4[1.2-3.0] | 2.7[1.8-3.8] | 0.8[0.7-1.6] |
| 6-month follow-up | 2.7[1.6-2.9] | 2.8[2.7-3.2] | 0.9[0.7-1.3] |
| P-value of baseline versus acutely  following CRT | 0.194 | 0.047 | 0.300 |
| P-value of baseline versus 6-months | 0.435 | 0.026 | 0.220 |
| P-value of acutely following CRT  versus 6-months | 0.433 | 0.502 | 0.759 |
|  |  |  |  |
| **Forward expansion wave (W/m^2^/s^2^×10^6^)** |  |  |  |
| Baseline before CRT | 0.3[0.3-0.9] | 0.3[0.3-0.6] | 0.9[0.5-0.9] |
| Acutely following CRT | 0.5[0.2-0.8] | 0.5[0.3-0.8] | 0.2[0.1-0.3] |
| 6-month follow-up | 0.3[0.2-0.6] | 0.5[0.3-0.9] | 0.2[0.2-0.2] |
| P-value of baseline versus acutely  following CRT | 0.807 | 0.120 | 0.109 |
| P-value of baseline versus 6-months | 0.859 | 0.547 | 0.285 |
| P-value of acutely following CRT  versus 6-months | 0.790 | 0.829 | 1.000 |
|  |  |  |  |
| **Backward compression wave (W/m^2^/s^2^×10^6^)** |  |  |  |
| Baseline before CRT | 0.3[0.1-0.4] | 0.2[0.1-0.3] | 0.3[0.3-0.7] |
| Acutely following CRT | 0.1[0.1-0.6] | 0.4[0.1-0.7] | 0.1[0.1-0.1] |
| 6-month follow-up | 0.3[0.2-0.3] | 0.2[0.2-0.3] | 0.1[0.1-0.1] |
| P-value of baseline versus acutely  following CRT | 0.701 | 0.123 | 0.109 |
| P-value of baseline versus 6-months | 0.086 | 0.657 | 0.109 |
| P-value of acutely following CRT  versus 6-months | 0.374 | 0.520 | 1.000 |

^†^Comparison between biventricular pacing and intrinsic rhythm at defined heart rate

Results are presented as median[interquartile range] for ease of comparison.

**Table A.3.** Invasive hemodynamic data

| **Variable** | **Overall (N=7)** | | | **CRT responder (N=4)** | | | **CRT non-responder (N=3)** | | |
| --- | --- | --- | --- | --- | --- | --- | --- | --- | --- |
|  | **INT** | **BVP** | ***P-*value^†^** | **INT** | **BVP** | ***P-*value^†^** | **INT** | **BVP** | ***P-*value^†^** |
| MAP, mmHg | 86±23 | 86±21 | 0.798 | 88±31 | 88±29 | 0.867 | 83±12 | 84±6 | 0.875 |
| SVR, mmHgmin/L | 29±16 | 21±8 | 0.122 | 37±17 | 23±8 | 0.049 | 17±2 | 19±9 | 0.760 |
| LVEDV, mL | 221±71 | 221±78 | 0.939 | 212±92 | 214±94 | 0.807 | 232±48 | 232±70 | 0.984 |
| LVESV, mL | 171±53 | 156±50 | 0.012 | 173±68 | 157±66 | 0.050 | 168±39 | 155±28 | 0.250 |
| SV, mL | 50±26 | 66±38 | 0.048 | 39±24 | 57±30 | 0.019 | 64±25 | 77±51 | 0.497 |
| CO, L/min | 4±2 | 5±3 | 0.079 | 3±2 | 5±3 | 0.018 | 5±1 | 5±3 | 0.648 |
| SW, mmHg.mL | 5132±2693 | 6326±3345 | 0.030 | 4558±2964 | 6131±3252 | 0.020 | 5899±2653 | 6586±4186 | 0.520 |
| EDPVR, mmHg/mL | 0.11±0.06 | 0.09±0.03 | 0.456 | 0.14±0.07 | 0.12±0.02 | 0.606 | 0.07±0.01 | 0.06±0.01 | 0.492 |
| ESPVR, mmHg/mL | 0.75±0.21 | 0.81±0.22 | 0.086 | 0.83±0.24 | 0.90±0.23 | 0.069 | 0.65±0.14 | 0.68±0.16 | 0.634 |
| LV dP/dt_max,_, mmHg/s | 855±140 | 918±194 | 0.144 | 862±194 | 991±240 | 0.011 | 846±40 | 820±40 | 0.615 |
| LV dP/dt_min_, mmHg/s | 840±104 | 860±147 | 0.699 | 900±59 | 960±67 | 0.014 | 760±101 | 726±105 | 0.794 |

^†^Comparison between BVP and INT.

BVP, biventricular pacing at baseline heart rates; CO, cardiac output; EDPVR, end-diastolic pressure-volume relationship; ESPVR, end-systolic pressure-volume relationship; INT, intrinsic rhythm at baseline heart rates; LV, left ventricular; LVEDV, left ventricular end-diastolic volume; LVESV, left ventricular end-systolic volume; MAP, mean arterial pressure; SV, stroke volume; SVR, systemic vascular resistance; SW, stroke work

Results are presented as mean±standard deviation.
