# Supplementary material for: The physiological effects of cardiac resynchronization therapy on aortic and pulmonary flow and dynamic and static components of systemic impedance
Source: Heart Rhythm O2. 2021 May 28;2(4):365–73. doi: 10.1016/j.hroo.2021.05.007 (PMC8369303; doi:10.1016/j.hroo.2021.05.007)
Supplement: Supplemental Appendix B [file mmc2.docx]

Appendix B

**Table B1. Invasive aortic wave intensity analysis after cardiac resynchronization therapy implant**

| **Variable** | **Overall** | **CRT responders** | **CRT non-responders** |
| --- | --- | --- | --- |
| **Forward compression wave (W/m^2^/s^2^×10^5^)** |  |  |  |
| Baseline before CRT | 4.4 [2.9-7.0] | 4.8 [3.0-7.0] | 3.8 [3.1-5.5] |
| Acutely following CRT | 5.3 [3.4-8.3] | 8.3 [4.4-8.4] | 3.9 [3.1-5.1] |
| P-value of baseline versus acutely  following CRT | 0.105 | 0.023 | 0.236 |

Results are presented as median [interquartile range] for ease of comparison.

**Table B2. Invasive pulmonary wave intensity analysis after cardiac resynchronization therapy implant**

| **Variable** | **Overall** | **CRT responders** | **CRT non-responders** |
| --- | --- | --- | --- |
| **Forward compression wave (W/m^2^/s^2^×10^5^)** |  |  |  |
| Baseline before CRT | 0.8 [0.7-1.3] | 1.2 [0.8-1.6] | 0.7 [0.6-0.8] |
| Acutely following CRT | 1.2 [0.7-1.3] | 0.8 [0.4-1.2] | 1.3 [1.2-1.6] |
| P-value of baseline versus acutely  following CRT | 0.633 | 0.004 | 0.183 |
|  |  |  |  |
| **Forward expansion wave (W/m^2^/s^2^×10^5^)** |  |  |  |
| Baseline before CRT | 0.3 [0.2-0.4] | 0.4 [0.4-0.5] | 0.2 [0.2-0.3] |
| Acutely following CRT | 0.1 [0.1-0.3] | 0.2 [0.1-0.3] | 0.1 [0.1-0.3] |
| P-value of baseline versus acutely  following CRT | 0.275 | 0.030 | 0.763 |
|  |  |  |  |
| **Backward compression wave (W/m^2^/s^2^×10^5^)** |  |  |  |
| Baseline before CRT | 0.04 [0.01-0.05] | 0.05 [0.05-0.07] | 0.01 [0.01-0.01] |
| Acutely following CRT | 0.01 [0.01-0.01] | 0.01 [0.01-0.04] | 0.01 [0.01-0.01] |
| P-value of baseline versus acutely  following CRT | 0.323 | 0.364 | 0.423 |

Results are presented as median [interquartile range] for ease of comparison.
